# Taxonomic and predicted functional signatures reveal linkages between the rumen microbiota and feed efficiency in dairy cattle raised in tropical areas

**DOI:** 10.3389/fmicb.2022.1025173

**Published:** 2022-11-29

**Authors:** Priscila Fregulia, Mariana Magalhães Campos, Roberto Júnio Pedroso Dias, Junhong Liu, Wei Guo, Luiz Gustavo Ribeiro Pereira, Marco Antônio Machado, Daniele Ribeiro de Lima Reis Faza, Le Luo Guan, Phil C. Garnsworthy, André Luis Alves Neves

**Affiliations:** ^1^Laboratório de Protozoologia, Instituto de Ciências Biológicas, Universidade Federal de Juiz de Fora, Juiz de Fora, Minas Gerais, Brazil; ^2^Programa de Pós-Graduação em Biodiversidade e Conservação da Natureza, Instituto de Ciências Biológicas, Universidade Federal de Juiz de Fora, Juiz de Fora, Minas Gerais, Brazil; ^3^Brazilian Agricultural Research Corporation (Empresa Brasileira de Pesquisa Agropecuária, EMBRAPA), National Center for Research on Dairy Cattle, Juiz de Fora, Minas Gerais, Brazil; ^4^Department of Agricultural, Food and Nutritional Science, University of Alberta, Edmonton, AB, Canada; ^5^Key Laboratory of Animal Genetics, Breeding and Reproduction in the Plateau Mountainous Region, Ministry of Education, Guizhou University, Guiyang, China; ^6^School of Biosciences, University of Nottingham, Loughborough, United Kingdom; ^7^Department of Veterinary and Animal Sciences, Faculty of Health and Medical Sciences, University of Copenhagen, Frederiksberg, Denmark

**Keywords:** RFI, functional microbial composition, rumen microbiome, SSU rRNA, taxonomic microbial composition

## Abstract

Ruminants digest plant biomass more efficiently than monogastric animals due to their symbiotic relationship with a complex microbiota residing in the rumen environment. What remains unclear is the relationship between the rumen microbial taxonomic and functional composition and feed efficiency (FE), especially in crossbred dairy cattle (Holstein x Gyr) raised under tropical conditions. In this study, we selected twenty-two F1 Holstein x Gyr heifers and grouped them according to their residual feed intake (RFI) ranking, high efficiency (HE) (*n* = 11) and low efficiency (LE) (*n* = 11), to investigate the effect of FE on the rumen microbial taxa and their functions. Rumen fluids were collected using a stomach tube apparatus and analyzed using amplicon sequencing targeting the 16S (bacteria and archaea) and 18S (protozoa) rRNA genes. Alpha-diversity and beta-diversity analysis revealed no significant difference in the rumen microbiota between the HE and LE animals. Multivariate analysis (sPLS-DA) showed a clear separation of two clusters in bacterial taxonomic profiles related to each FE group, but in archaeal and protozoal profiles, the clusters overlapped. The sPLS-DA also revealed a clear separation in functional profiles for bacteria, archaea, and protozoa between the HE and LE animals. Microbial taxa were differently related to HE (e.g., *Howardella* and *Shuttleworthia*) and LE animals (e.g., *Eremoplastron* and *Methanobrevibacter)*, and predicted functions were significatively different for each FE group (e.g., K03395—signaling and cellular process was strongly related to HE animals, and K13643—genetic information processing was related to LE animals). This study demonstrates that differences in the rumen microbiome relative to FE ranking are not directly observed from diversity indices (Faith’s Phylogenetic Diversity, Pielou’s Evenness, Shannon’s diversity, weighted UniFrac distance, Jaccard index, and Bray–Curtis dissimilarity), but from targeted identification of specific taxa and microbial functions characterizing each FE group. These results shed light on the role of rumen microbial taxonomic and functional profiles in crossbred Holstein × Gyr dairy cattle raised in tropical conditions, creating the possibility of using the microbial signature of the HE group as a biological tool for the development of biomarkers that improve FE in ruminants.

## Introduction

Feed fermentation in the rumen is powered by the activity of a vast array of anaerobic microbes that live in symbiosis with the host animal (Oliveira et al., 2007). These microbes comprise representative taxa of prokaryotic (bacteria and archaea) and eukaryotic (fungi and protozoa) organisms. Bacteria are the most abundant rumen microorganisms, playing an essential role in the degradation of plant fiber and starch (Moraïs and Mizrahi, 2019). Archaea, mainly constituted of methanogens, reduce CO_2_ to CH_4_ to maintain a low hydrogen pressure in the rumen (Bodas et al., 2012). Fungi are powerful fiber digesters, penetrating both the cuticle and the cell wall of lignified materials (Moraïs and Mizrahi, 2019). Rumen protozoa account for up to 50% of the microbial biomass and are responsible for predating bacteria and enhancing methanogenesis in association with archaea (Morgavi et al., 2010). Despite the importance of protists for the rumen environment, few studies have attempted to understand the relationship between rumen protozoa and feed efficiency (FE) (Zhang et al., 2020; Clemmons et al., 2021).

Rumen microbes are believed to affect the FE of the host, and this effect has been observed mainly when residual feed intake (RFI) is used as the FE measurement (Guan et al., 2008; Auffret et al., 2020) and also when FE has been measured directly as feed conversion ratio (Roehe et al., 2016). Studies correlating the rumen microbiome with RFI have predominantly been developed in cattle raised in temperate climates (Auffret et al., 2020; Welch et al., 2021). However, little is known about the linkage between the rumen microbiome and RFI in breeds raised in tropical regions. In addition to the diet, the composition of the rumen microbiome is affected by the breed (Li et al., 2019a) and the environmental conditions (e.g., temperature) where the animal is raised (Dehority and Orpin, 1997). Rumen function and fermentation can be affected when temperate climate breeds are exposed to high atmospheric temperatures in tropical areas (Baumgard et al., 2007; Passini et al., 2009), indicating that both the microbiome and FE are altered in animals raised under heat stress.

Studies on the relationship between the rumen microbiome and RFI have not reached a consensus (Auffret et al., 2020; Bowen et al., 2020; Fregulia et al., 2021). Some authors say that the relationship between the rumen microbiome and RFI phenotype may not be explained at the community level because of the redundant role the microbial taxa play in the rumen function (Fregulia et al., 2021). Other authors report that the functional profile of the rumen microbiota is more related to FE than the taxonomic profile itself (Clemmons et al., 2019; Li et al., 2019b). Shabat et al. (2016) and Li and Guan (2017) found that efficient cattle had a lower number of biochemical-enriched pathways than their inefficient counterparts, suggesting that the rumen microbiome of efficient cattle has more restricted metabolic pathways and maintains only those functions that are relevant to the health and productive traits of the host animal. The current study investigated the effect of RFI phenotype on rumen microbial taxa (bacteria, archaea, and protozoa) and their functions in dairy cattle raised under tropical conditions, with the hypothesis that the relationship between feed efficiency and the rumen microbiome is influenced by the breed and the environment where the animal is raised.

## Materials and methods

All experimental procedures involving animals in this study were approved by the Ethics Committee of Embrapa Dairy Cattle (number: 05/2015). This study was performed at the Multi-use Complex on Livestock Bioefficiency and Sustainability of Brazilian Agricultural Research Corporation (Embrapa), located in Coronel Pacheco, Minas Gerais, Brazil.

### Animal experiments and sample collection

This experiment was conducted as part of a larger study designed to examine the biological parameters in heifers F1 Holstein × Gyr related to feed efficiency (Leão et al., 2018; Ornelas et al., 2019; Cabral da Silva et al., 2020; Fonseca et al., 2020; Marçal-Pedroza et al., 2020). A detailed description of the performance data, calculation of FE indices, and group classifications are provided by Cabral da Silva et al. (2020). Briefly, 22 F1 Holstein × Gyr heifers were used in this study, averaging (mean ± SD) 258 ± 20 days of age and 293 ± 21.5 kg body weight (BW) recorded at the beginning of the metabolism study. Heifers were housed in individual tie stalls (2.5 × 1.2 m) with bedding made of rubber mats (WingFlex, Kraiburg TPE GmbH & Co., Waldkraiburg, Germany). The average temperature and precipitation during the experimental period were 22°C and 0.019 mm/day (Instituto Nacional de Meteorologia [INMET], 2015). The experimental diet was formulated to meet the nutritional requirements of the heifers following the guidelines of the NRC Dairy Cattle (National Research Council [NRC], 2001). Dry matter (DM) and crude protein (CP) contents of the experimental diet were 43.8% and 175 g/kg DM, respectively. The diet included (DM basis) 75% corn silage and 25% concentrate [96% soybean meal and 4% mineral premix (Fosbovi 40, MN, DSM1 São Paulo/Brazil), DM basis]. The daily offered amount of the total mixed ration was adjusted to allow 10% orts on an as-fed basis, according to the intake observed on the previous day. Animals were evaluated according to the RFI index and classified into two groups: (1) high efficiency (HE) (RFI –0.2600 ± 0.05) and (2) low efficiency (LE) (RFI 0.3229 ± 0.17), with 11 animals per group. Rumen fluids were collected using a stomach tube with a rumen vacuum sampler, snap-frozen using liquid nitrogen, and stored below –80°C for further analysis. In this experiment, the stomach tube was inserted at the appropriate depth (120–250 cm) to reach the central rumen sites and reduce saliva contamination. Additionally, we discarded the first 30 ml of ruminal contents present in the tube after sample collection and washed the probe thoroughly before using it to collect rumen samples between the current animal and the next one.

### DNA extraction, library preparation, and sequencing

Total DNA was extracted from 800 μl of each rumen fluid sample using bead-beating and phenol-chloroform extraction methods (adapted from Oliveira et al., 2007). Briefly, 800 μl of rumen fluid sample were transferred to a new tube and washed with 1 mL of lysis buffer (500 mM NaCl; 50 mM Tris-HCl, pH 8.0, 50 mM EDTA, 4% SDS). Subsequently, 2 μl RNase were added to each sample, and tubes were incubated at 37°C for 15 min. Then 20 μl of proteinase K were added to each tube, and cells were lysed by physical disruption using bead beating with a BioSpec Mini Bead-Beater (BioSpec, Bartlesville, OK, USA) at 4,800 rpm for 4 min. The tubes were incubated at 70°C for 15 min. The supernatant obtained from each sample was transferred to a new tube for subsequent phenol-chloroform-isoamyl alcohol extraction. Extracted DNA was precipitated with ammonium acetate 10M and cold 100% isopropanol. After 30 min in the freezer, tubes were centrifuged at 16,000 × g for 10 min, and supernatant was removed. Cold 70% ethanol was added to each sample, and the tubes were centrifuged at 16,000 × g for 2 min. The supernatant was removed, and the remaining content was resuspended in 200 μl of buffer TE (10 mM Tris; 1 mM EDTA). All samples were analyzed using both NanoDrop spectrophotometer (NanoDrop Technologies, Inc., Wilmington, DE) and Qubit Quantification Platform (Invitrogen Ltd., Paisley, UK) to accurately assess DNA quantity and quality.

Amplicon library preparation (*n* = 22) was performed by PCR amplification of the V4 region of the 16S rRNA gene of bacteria and archaea, using the primers 515F (5s-Adaptor/GTGCCAGCMGCCGCGGTAA) and 806R (5G-Adaptor/GGACTACHVGGGTWTCTAAT) (Caporaso et al., 2011); and by amplification of the V3-V4 region of the 18S rRNA gene of protozoa, using the primers 316F (5s-Adaptor/GCTTTCGWTGGTAGTGTATT) and 539R (5C-Adaptor/CTTGCCCTCYAATCGTWCT) (Sylvester et al., 2004). Illumina TruSeq libraries were prepared and paired-sequenced (2 × 250 bp) on an Illumina HiSeq 2500 sequencing platform (Illumina, Inc., San Diego, CA, USA). The data presented in the study are deposited in the NCBI’s Sequence Read Archive, under the BioProject PRJNA893629.

### Amplicon sequencing data analysis

Bioinformatics analysis followed the procedure described by Liu (2020). In detail, sequencing data were analyzed using Quantitative Insights Into Microbial Ecology 2 (QIIME 2) version 2020.8 (Bolyen et al., 2019). The demultiplexed raw sequence data files were imported into QIIME 2 using the “SampleData [PairedEndSequencesWithQuality]” semantic type. Data were demultiplexed (with parameter Phred33), and sequence reads were quality-filtered using the Divisive Amplicon Denoising Algorithm (DADA2) plugin implemented in QIIME2, with quality filtering Q-score > 25. After inspecting the interactive quality plot, we observed that the Q-score of the bases dropped off around position 180, and thus our sequences were truncated at 180 bases to remove low-quality regions of the sequences. To achieve this goal, DADA2 was used to denoise sequences with the truncation length parameters of *–p-trunc-len-f 180*, and *–p-trunc-len-r 180*. The sequences were merged, and chimeric sequences were removed before the generation of a table of amplicon sequencing variants (ASV) (Callahan et al., 2016). Representative sequences were aligned to the SILVA 132 Small Subunit rRNA Database for bacteria, using the database file “Silva 138 99% OTUs from 515F/806R region of sequences” available on the section QIIME2 docs, on the QIIME2 website. The classifier was pre-trained on the Silva 18S rRNA database (release 132) for protozoa and on the Rumen and Intestinal Methanogens Database (RIM-DB) for archaea (Quast et al., 2012; Seedorf et al., 2014a), using the *fit-classifier-naive-bayes* method implemented in the *q2-feature classifier* plugin.

### Predicting functional profile

Microbial functions were predicted by reconstructing the unobserved states for 16S and 18S rRNA gene sequences. The tool Phylogenetic Investigation of Communities by Reconstruction of Unobserved States 2 (PICRUSt2) implemented in QIIME2 (Douglas et al., 2020) is based on the Integrated Microbial Genomes (IMG) database (Markowitz et al., 2012) and was used to predict MetaCyc pathways for bacterial, archaeal, and protozoal ASV (Caspi et al., 2014).

### Statistical analysis

To analyze microbial diversity among samples, sequence counts of all samples were standardized by rarefying them to the same number of sequences (the smallest sampling size) using the *q2-feature-table*. The plugin *q2-diversity* then used the rarefied feature table and the phylogenetic tree to calculate diversity metrics. To investigate alpha-diversity metrics, Faith’s PD, Pielou’s evenness, and Shannon’s entropy were calculated. To investigate beta-diversity metrics, weighted UniFrac distance, Jaccard index, and Bray–Curtis dissimilarity index were calculated. Samples were rarified to 39,605 sequences for 16S rRNA and 165,430 sequences for 18S rRNA. Dissimilarity and distance between the rumen microbiota and the categorical RFI groups were tested using unweighted UniFrac distance matrices and Permutational Multivariate Analysis of Variance (PERMANOVA) with 999 permutations. Based on this analysis, plots were generated using the visualizer tool of the *q2-diversity* plugin.

Finally, the mixMC multivariate method implemented in the *mixOmics* R package was used to identify associations between microbial profiles and functions (microbial and functional signatures) and HE and LE animals. For this analysis, only microbial taxa and microbial functions with relative abundance > 0.01% and prevalence in at least 50% of samples were considered (11 out of 22). Then, sparse partial least square discriminant analysis (sPLS-DA) (Lê Cao et al., 2016) was applied to identify microbial signatures related to HE and LE animals.

## Results

### Sequencing information

A total of 2,132,659 16S rDNA gene reads and 7,084,856 18S rDNA gene reads were generated from rumen samples collected from the 22 animals. After quality control, combining paired-end reads, and filtering chimeras, on average, 91% of sequences passed the filters, with 1,661,299 sequences separated as 16S rDNA and 6,111,590 as 18S rDNA. An average of 75,513 (± 12,226) denoised sequences were generated per animal for 16S rRNA and 277,799 (± 58,359) for 18S rRNA. Good’s coverages for both 16S and 18S rRNA were higher than 98%, suggesting that sequencing depth had sufficient coverage for the microbial communities.

### Microbial community structure

Bacterial, archaeal, and protozoal community structure was examined using Faith’s PD, Pielou’s evenness, and Shannon’s entropy. None of these alpha-diversity metrics was different between HE and LE animals. Additionally, beta-diversity metrics, such as unweighted UniFrac, did not differ between HE and LE animals (Supplementary Table 1). Weighted UniFrac distances showed no differences in the structuring of the microbial communities according to the feed efficiency groups. As for the Jaccard index, the communities overlapped, indicating that the structures of the microbial communities from both groups were similar. The Bray–Curtis dissimilarity matrix showed no microbial cluster by feed efficiency grouping (data not presented).

### Taxonomic profile

**FIGURE 1:**
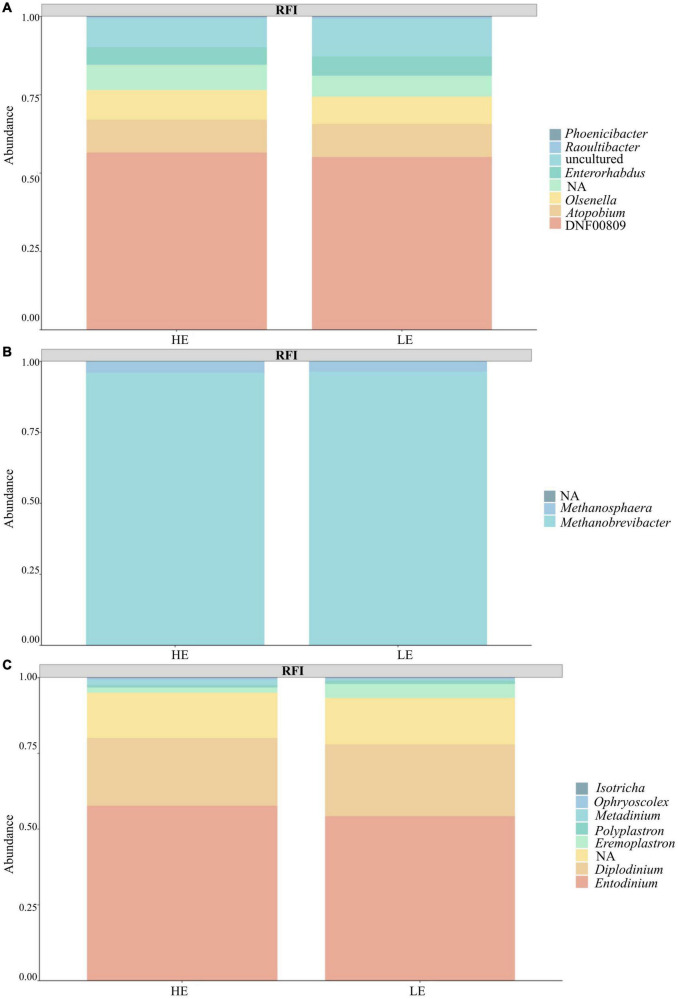
Taxa summary plot between the feed efficiency groups (FE and LE). **(A)** Bacteria. **(B)** Archaea. **(C)** Protozoa.

Taxonomic profiling revealed a total of 22 prokaryotic taxa at the phylum level and eight eukaryotic taxa at the phylum level. From 16S rRNA gene sequences, after filtration, 74% species belonged to the Bacteria kingdom, 24% to Archaea, and 2% were unclassified. The dominant prokaryotic phylum was *Firmicutes* (52%), followed by *Euryarchaeota* (24%) and *Bacteroidota* (18%). At the genus level, the predominant taxa were *Methanobrevibacter* (23%), *Christensenellaceae* R7 group (10%), and *Prevotella* (8%). From 18S rRNA gene sequences, 95% were classified as Protozoa, < 0.001% were classified as Fungi, and 5% were unclassified. The dominant phylum was Ciliophora (95%), followed by an unassigned group (5%). At the genus level, the predominant taxa were *Entodinium* (53%), *Diplodinium* (22%), and an unassigned group (15%). Notably, 14% of 16S rRNA sequences and 20% of 18S rRNA sequences could not be assigned to a known genus (Figure 1).

In order to better represent the taxonomic and predicted functional profile of the rumen microbiota related to RFI, all detected taxa, including unclassified taxa, were included in the analysis. Fungi taxa sequenced by 18S rRNA primer were removed from the analysis because this molecular marker is unsuitable for fungi classification due to low-quality resolution. A complete list of all microbial taxa is in Supplementary Table 2.

### Predicted functional profile

Using the PICRUSt2 package in QIIME2, a total of 6,774 and 7,636 MetaCyc pathways were predicted based on 16S rRNA and 18S rRNA, respectively. A complete list of all predicted MetaCyc pathways is provided in Supplementary Table 3.

Metabolic pathways were predicted for bacteria and archaea separately, but the RIM-DB was used to improve the classification of archaea, generating a new and improved dataset for this microbial group. Even with these two datasets (bacteria and archaea) being used as inputs for PICRUSt2, MetaCyc pathways predicted for both datasets were the same, with identical frequency per feature. The ten most abundant pathways reconstructed for each microbial group were considered the major predicted functions of the rumen microbiome in crossbred Holstein x Gyr dairy cattle (Supplementary Table 4).

### Taxonomic and functional signatures related to residual feed intake

**FIGURE 2:**
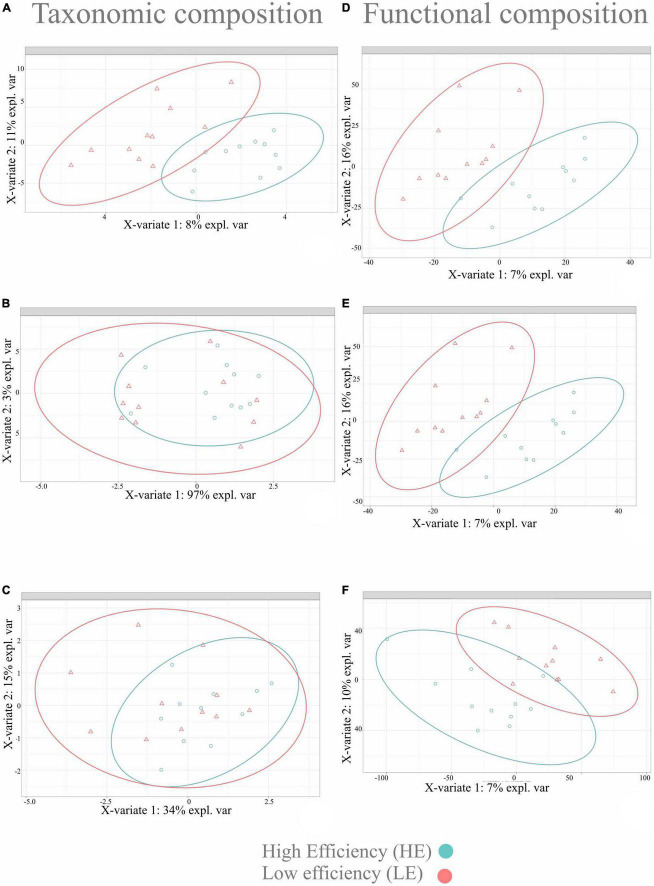
Sparse partial least square discriminant analysis results on rumen microbiome in two FE groups of dairy cattle. Sample plot on the two first sPLS-DA components with 95% confidence level ellipse plots. **(A)** Bacterial taxonomic composition; **(B)** archaeal taxonomic composition; **(C)** protozoal taxonomic composition; **(D)** bacterial functional composition; **(E)** archaeal functional composition; **(F)** protozoal functional composition.

The sPLS-DA multivariate analysis was used to identify microbial taxa and functions that best characterized the HE and LE animals. For this analysis, only microbial taxa and functions with a relative abundance > 0.01% and prevalent in at least 50% of the samples (11 out 22) were considered (Supplementary Figure 1). Twenty-one phyla and 49 genera of bacteria and archaea were detected, and two phyla and seven genera of protozoa were detected. After pre-training the classifier to improve archaeal classification, a new dataset was generated for only archaea, with one phylum and two genera. Following centered log-ratio transformation procedures, a clear separation was observed in the bacterial taxonomic profile differentiating the rumen microbiome of HE from LE. Still, no difference was observed for archaeal and protozoal taxonomic profiles (Figure 2). However, a clear separation of functional profiles for bacteria, archaea, and protozoa was observed between HE and LE animals (Figure 2).

**FIGURE 3:**
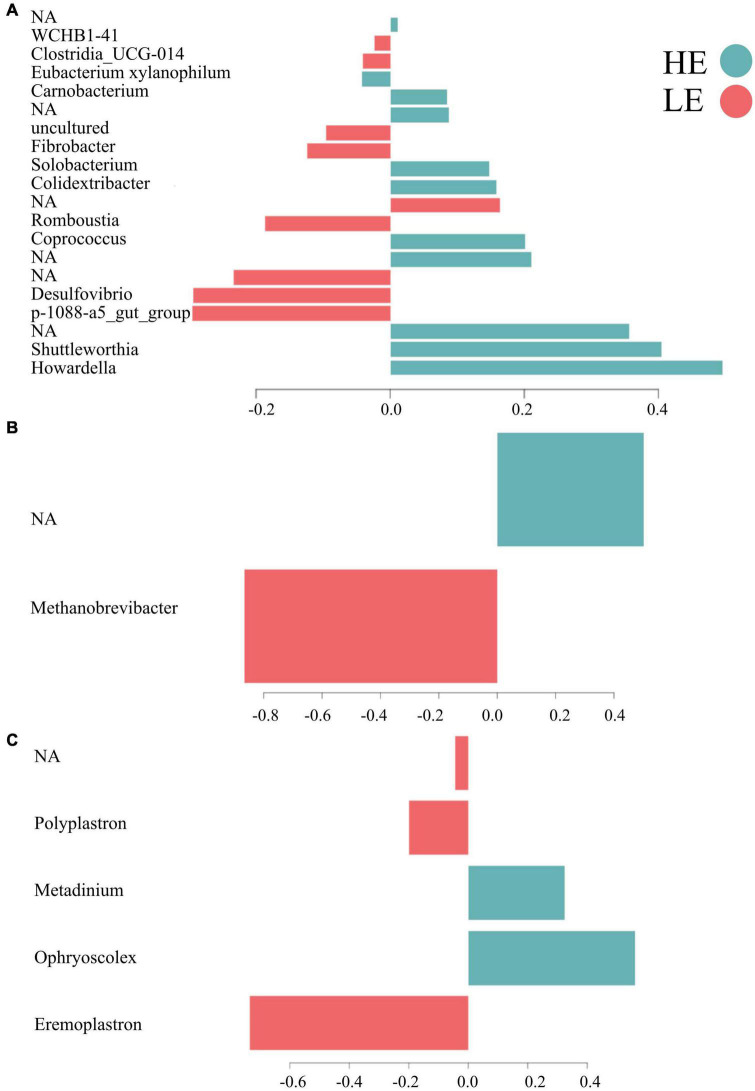
Contribution of each microbial taxa selected on the first component. **(A)** Bacteria. **(B)** Archaea. **(C)** Protozoa.

Overall, 55% of the bacterial signature selected in component 1 of the sPLS-DA characterized the rumen microbiome of HE animals, which included the bacterial taxa *Howardella*, *Shuttleworthia*, *Coprococcus*, *Colidextribacter*, *Solobacterium*, *Carnobacterium*, [*Eubacterium*] *xylanophilum* group, and four unclassified taxa. From the new dataset generated for archaea, 50% of the archaeal signature selected in component 1 characterized the rumen microbiome of HE animals, having members of the taxa *Methanobrevibacter* and of one unclassified taxon. On the other hand, 60% of the protozoal signature selected on component 1 of the sPLS-DA characterized the rumen microbiome of LE animals and comprised the taxa *Eremoplastron*, *Polyplastron*, and one unclassified taxon (Figure 3).

**FIGURE 4:**
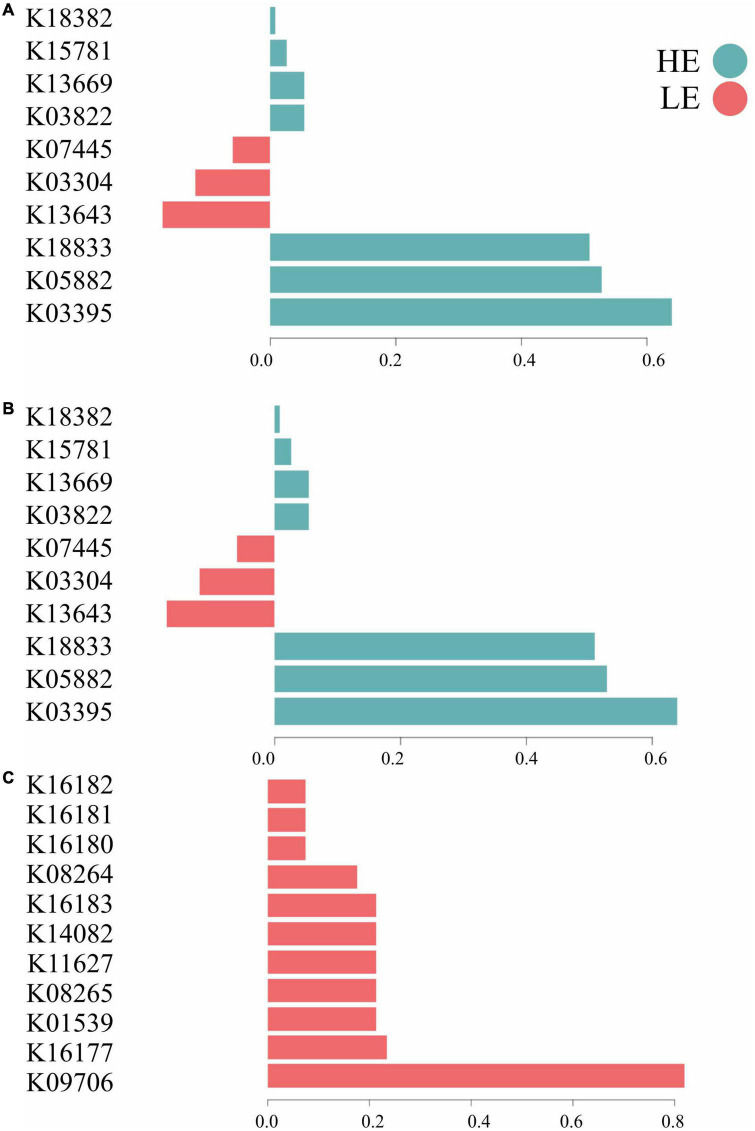
Contribution of each microbial function selected on the first component. **(A)** Bacterial function; **(B)** archaeal function; **(C)** protozoal function.

The most important MetaCyc pathways for component 1 in bacteria and archaea were related to metabolism (50%), signaling and cellular processes (30%), and genetic information processing (20%). For protozoa, the most important MetaCyc pathways on component 1 were related to metabolism (72%), environmental information processing (9%), genetic information processing (9%), and unknown functions (9%) (Figure 4). In terms of functional signature, 70% of the bacterial and archaeal signatures selected in component 1 of the sPLS-DA characterized the rumen MetaCyc pathways of HE animals, including functions related to signaling and cellular processes (e.g., K03395, K18833, and K03304), metabolism (e.g., K05882, K03822, K13669, K15781, K18382) and genetic information processing (e.g., K13643 and K07445). For protozoa, 100% of the signature selected on this same component of the sPLS-DA characterized the rumen MetaCyc pathways of LE animals, including functions related to metabolism (e.g., K16177, K08265, K14082, K16183, K08264, K16180, K16181, and K16182), environmental information processing (e.g., K01539), genetic information processing (e.g., K11627) and unknown functions (e.g., K09706) (Figure 4).

## Discussion

To our knowledge, this is the first report that documents the linkage between the rumen microbiota and its functions and the RFI phenotype in dairy cattle raised in tropical conditions. First, alpha- and beta-diversity indices did not differ between the HE and LE animals, and in agreement with previous studies on breeds raised in temperate climates (Myer et al., 2015; Clemmons et al., 2019), these findings suggest that diversity indices may not be a significant parameter to differentiate feed efficiency phenotypes. Second, microbial signatures are more useful than diversity indices for detecting correspondences between specific taxa and RFI phenotypes in ruminants (Shabat et al., 2016; Delgado et al., 2019). Third, MetaCyc pathways predicted from PICRUSt2 and analyzed through mixMC could separate functional microbial profiles related to RFI for bacteria, archaea, and protozoa and identify specific metabolic pathways associated with HE and LE animals. In line with Shabat et al. (2016), our findings suggest that the functional profile of the rumen microbiota can be more informative about FE phenotypes than the taxonomic profile of the whole microbial community.

The lack of difference in alpha- and beta-diversity indices indicates that RFI phenotypes cannot be a reflection of the diversity of the microbial community but the result of dissimilarities at a finer resolution at the species and/or genus level of the microorganisms and their functions (McGovern et al., 2020). Microorganisms belonging to different taxonomic groups may play the same role in the rumen, utilizing similar substrates and producing similar products (Clemmons et al., 2019; Li et al., 2019a). This may indicate that detecting specific microbial taxa and their functions is fundamental to understanding the linkage between the RFI phenotype and the taxonomic structure of the rumen microbiome. Studies that used PCR-DGGE to understand the linkage between microbial community structure and FE have reported that bacterial profiles generated from LE animals were grouped together and separated from profiles obtained from HE animals (Guan et al., 2008). In the current study, the sPLS-DA models showed a clear separation in bacterial and archaeal profiles differentiating the HE and LE animals, but not protozoal profiles (Figure 1). Carberry et al. (2012) found that diet influenced the effect of RFI on bacterial profile, especially when animals were fed on a forage-based diet. This is in agreement with our results, as our animals were fed on forage-based diets in a similar fashion. Nevertheless, most recent studies have suggested that specific microbial taxa, and not the whole rumen microbiome, could be the main agents driving differences in FE phenotypes (Carberry et al., 2012; Elolimy et al., 2018; Brooke et al., 2019).

The microbial signatures identified in this study provide further understanding of the relationships between RFI and the rumen microbiota and its functions, demonstrating the feasibility of probing rumen microbial signatures with sPLS-DA models (Lê Cao et al., 2016; Neves et al., 2020) to differentiate FE phenotypes. The bacterial signature of HE animals included members of the families *Lachnospiraceae* (*Howardella*, *Shuttleworthia*, *Coprococcus*, and *Eubacterium xylanophilum*), *Oscillospiraceae* (*Colidextribacter*), *Erysipelotrichaceae* (*Solobacterium*), *Carnobacteriaceae* (*Carnobacterium*), and four unidentified taxa (Figure 2). The genus members of *Lachnospiraceae* have been previously related to feed efficiency in cattle (Jewell et al., 2015; Shabat et al., 2016; Elolimy et al., 2018; Wallace et al., 2019) as well as in other animals, such as pigs and chickens (Lee et al., 2017; Aliakbari et al., 2021). Among all genera described above, the following three genera are the only ones with known functions in the rumen, suggesting that more research is needed on the functional role of the unknown microbes. *Howardella* plays a role in urea hydrolysis (Cook et al., 2007). The ureolytic bacteria are the most important organisms associated with N metabolism in the rumen, enabling the breakdown of urea to ammonia used for the biosynthesis of microbial protein for the host (Hailemariam et al., 2021). *Shuttleworthia* participates in lipid and carbohydrate metabolism and regulates the endocrine system *via* short-chain fatty acid production, potentially increasing the host feed efficiency (Liu et al., 2021). *Coprococcus* has been extensively related to high feed efficient cattle (Jewell et al., 2015; Shabat et al., 2016) and plays a role in metabolizing carbohydrates for the host (Whitman et al., 2015). These findings suggest that the same microbial taxa can influence the feed efficiency of different host organisms, highlighting the necessity of deepening our understanding of the functional role of the diverse microbial taxa present in the rumen.

The bacterial signature of the LE group included members of the families *Pirellulaceae* (p-1088-a5 gut group), *Desulfovibrionaceae* (*Desulfovibrio*), *Peptostreptococcaceae* (*Romboutsia*), *Fibrobacteraceae* (*Fibrobacter*), *Clostridia* UCG-014, WCHB1-41, and three unidentified taxa. Although *Pirellulaceae* p-1088-a5 gut group was associated with inefficient cattle in this study, it has been related to feed efficient pigs (Gardiner et al., 2020) and calcium digestibility in goats (Liu et al., 2020), indicating that our findings may not be conclusive evidence to support this association. *Desulfovibrio* is responsible for removing toxic hydrogen sulfide gas from the rumen when ruminants ingest high sulfate concentrations. Hydrogen sulfide can inhibit the production of volatile fatty acids, especially butyrate, decreasing feed efficiency (Zhang et al., 2021). *Romboutsia* is related to less severe immune responses, as demonstrated by reduced concentrations of pro-inflammatory plasma cytokines (Liang et al., 2016) measured in inefficient animals that exhibited downregulated immune functions (Kern et al., 2016). These findings reveal that the functions of most rumen microorganisms are not well understood, and more metatranscriptomics studies are needed to elucidate the functional landscape of the rumen microbiome.

Unfortunately, the archaeal signature of HE animals was composed of unclassified taxa and could not be defined. This may be attributed to the fact that this study did not use archaea-specific primers, limiting the taxonomic assessment of this microbial group. Myer et al. (2016) also found many unassigned taxa that could be the key to understanding feed efficiency in ruminants. Projects, such as the Hungate 1000^1^ are crucial to investigating the rumen microbiome and relationships between archaeal taxa and feed efficiency. Exploring big data, like that of the Hungate 1000, might be the direction to uncovering novel archaeal signatures of feed-efficient animals. However, the archaeal signature of LE in this study was entirely composed of *Methanobrevibacter*, which accounts for the majority of rumen methanogens found in cattle. This genus is more abundant in inefficient cattle and is associated with higher enteric methane emissions (Delgado et al., 2019).

The protozoal signature comprised members of only one family, Ophryoscolecidae. In HE animals, this signature included members of the genera *Ophryoscolex* and *Metadinium*, and in LE, it included members of *Eremoplastron*, *Polyplastron*, and one genus not identified. Rumen protozoa play a significant role in microbial protein synthesis and nitrogen balance in the rumen (Morgavi et al., 2010; Guyader et al., 2014; Newbold et al., 2015). Unlike our results, previous studies detected differential abundance in *Diplodinium* and *Entodinium* genera in divergent FE groups (Zhang et al., 2020; Clemmons et al., 2021). However, the current study sheds light on the functional role of *Ophryoscolex* and *Metadinium* in feed efficiency that has not been previously observed or anticipated, despite the limited number of protozoan signatures detected. This information is far from complete and is insufficient to understand the complex relationships between rumen protozoa and feed efficiency because many species of these two genera still need to be characterized, indicating the need for further studies of their functions.

The rumen microbiome comprises an active community that lies at the interface of the animal and its environment, with microbial activity influencing the metabolism and physiology of the host animal (Ha et al., 2014). There is also evidence that the genetics of the host animal shapes a core rumen microbiome that is linked to feed efficiency (Li et al., 2019a; Wallace et al., 2019). Altering rumen microbial functions to enhance nutrient utilization may improve feed efficiency (Li and Guan, 2017). To understand the functional role of the whole microbial population without the need for culture, MetaCyc pathways were predicted in our samples using the tool PICRUSt2. MetaCyc is a database of metabolic pathways from all domains of life (Caspi et al., 2008). PICRUSt2 is a cost-effective method to predict functional abundances based only on marker gene sequences. Unlike metatranscriptomic analysis, it does not provide direct information about active genes in the microbial community. These approaches (PICRUSt2 and metatranscriptomics) are closely related in their predictive power and have been widely used to profile the functional features of the rumen microbiome (Neves et al., 2017; Douglas et al., 2020). When used correctly and appropriately, PICRUSt2 can profile 16S and 18S RNA genes with high reliability (Douglas et al., 2018; Wilkinson et al., 2018), as was done in this study. However, PICRUSt2 has limitations in identifying microbiome functions (Huws et al., 2018), and it is generally recommended to use metatranscriptomics instead of PICRUSt2 to obtain a more accurate description of the active microbiota.

The MetaCyc pathways predicted through the PICRUSt2 tool showed a clear separation in microbial functions of bacteria, archaea, and protozoa related to each FE group. In bacteria and archaea, 48 and 45%, respectively, of the metabolic pathways in HE animals were associated with various metabolism functions (e.g., carbohydrates and lipids). In protozoa, 36% of the MetaCyc pathways related to LE animals were associated with energy and amino acid metabolism, indicating that, unlike bacterial metabolic functions, protozoal metabolic pathways may be detrimental to feed efficiency (Figure 3). Our results agree with Shabat et al. (2016) and Li and Guan (2017), who showed that inefficient cattle display more diverse activities of rumen microbes than their efficient counterparts. According to Shabat et al. (2016), efficient cattle have simpler metabolic pathway networks than inefficient cattle, which may result in higher concentrations of products that are more relevant to the host, supporting a greater energy harvest efficiency. Changes in functional groups (e.g., a switch from proteolytic to saccharolytic fermentation) have been associated with higher energy harvest from feed and metabolic diseases in obese humans (Canfora et al., 2019). This host-microbe association suggests that microbial functions are of paramount importance to efficient digestion and absorption of nutrients in the gastrointestinal tract of mammals, pointing to the need to uncover novel microbial functions to understand better their influence on animal metabolism and phenotypes, such as FE.

## Conclusion

This study revealed compositional differences in specific taxa and MetaCyc pathways related to RFI phenotypes in dairy cattle raised in tropical conditions, but several taxa were unassigned when we profiled the microbial community at more specific levels (e.g., genus, ASV).

This article suggests that discovering biomarkers for FE phenotypes could be accomplished by identifying specific microbial taxa and metabolic pathways that characterize LE and HE groups. In this way, specific microbes and metabolic pathways could be manipulated in the rumen to improve FE. Additionally, we suggest that meta-omics data (e.g., metagenomics, metatranscriptomics, metabolomics) be incorporated in future studies to facilitate the discovery of biomarkers and provide a better overview of the rumen microbiome functionality and its association with FE phenotype.

## Data availability statement

The datasets presented in this study can be found in online repositories. The names of the repository/repositories and accession number(s) can be found in the article/ Supplementary material.

## Ethics statement

This animal study was reviewed and approved by the Ethics Committee of Embrapa Dairy Cattle (Number: 05/2015).

## Author contributions

MC, RD, MM, and LP conceived and designed the experiment. MC collected the samples. PF and DF extracted DNA from the samples. PF, JL, and AN ran the bioinformatic pipeline. PF, WG, and AN performed the statistical analysis. PF, MC, RD, and AN executed the experiment and wrote the manuscript. PG and LG contributed to the investigation and revised the manuscript. All authors read and approved the final version of the manuscript.

## Footnotes

1
http://www.rmgnetwork.org/hungate1000.html
