# Supplementary material for: Taxonomic and predicted functional signatures reveal linkages between the rumen microbiota and feed efficiency in dairy cattle raised in tropical areas
Source: Front Microbiol. 2022 Nov 29;13:1025173. doi: 10.3389/fmicb.2022.1025173 (PMC9745175; doi:10.3389/fmicb.2022.1025173)
Supplement: Supplementary file 1 [file Data_Sheet_1.docx]

**Supplementary Material**

**Taxonomic and predicted functional signatures reveal linkages between the rumen microbiota and feed efficiency in dairy cattle raised in tropical areas**

Priscila Fregulia^a,b^, Mariana Magalhães Campos^c^, Roberto Júnio Pedroso Dias^a,b^, Junhong Liu^d^, Wei Guo^e^, Luiz Gustavo Ribeiro Pereira^c^, Marco Antônio Machado^c^, Daniele Ribeiro de Lima Reis Faza^c^, Le Luo Guan^d^, Phil C. Garnsworthy^f^, André Luis Alves Neves^g*^

^a^Laboratório de Protozoologia, Instituto de Ciências Biológicas, Universidade Federal de Juiz de Fora, 36036-900, Juiz de Fora, Minas Gerais, Brazil

^b^Programa de Pós-graduação em Biodiversidade e Conservação da Natureza, Instituto de Ciências Biológicas, Universidade Federal de Juiz de Fora, Juiz de Fora, Brazil

^c^Brazilian Agricultural Research Corporation (Empresa Brasileira de Pesquisa Agropecuária, EMBRAPA), National Center for Research on Dairy Cattle, Juiz de Fora, Minas Gerais, 36038-330, Brazil

^d^Department of Agricultural, Food and Nutritional Science, University of Alberta, Edmonton, AB, Canada

^e^Key Laboratory of Animal Genetics, Breeding and Reproduction in the Plateau Mountainous Region, Ministry of Education, Guizhou University, Guiyang, China

^f^School of Biosciences, Sutton Bonington Campus, University of Nottingham, Loughborough LE12 5RD, United Kingdom

^g^Department of Veterinary and Animal Sciences, Faculty of Health and Medical Sciences, University of Copenhagen, Grønnegårdsvej 3, DK-1870, Frederiksberg C, Denmark

^*^Corresponding author: [andre.neves@sund.ku.dk](mailto:andre.neves@sund.ku.dk)

**Supplementary Tables**

**Supplementary Table S1.** Alpha-diversity and Beta-diversity statistics of the rumen microbiota in HE and LE dairy cattle. Significance determined at p ≤ 0.05.

| Diversity metric | Bacteria | Archaea | Protozoa |
| --- | --- | --- | --- |
|  | *P*-value | *P*-value | *P*-value |
| Faith’s Phylogenetic Diversity | 0.12 | 0.09 | 0.10 |
| Simpson’s Evenness | 0.71 | 0.66 | 0.92 |
| Good’s coverage | 98% | 98% | 99% |
| Unweighted UniFrac | 0.98 | 0.82 | 0.63 |

**Supplementary Table S2.** A complete list of bacteria, archaea and protozoa classified by Silva and RIM-DB in dairy cattle F1 Holstein x Gyr classified into two feed efficiency groups. Each microbial group is shown in a tab of the table.

**Supplementary Table S3.** A complete list of predicted MetaCyc pathways based on 16S rRNA and 18S rRNA from the rumen of dairy cattle F1 Holstein x Gyr classified into two feed efficiency groups. The dataset of each molecular marker is shown in a tab of the table.

**Supplementary Table S4.** The main predicted microbial functions on rumen of dairy cattle divergent to feed efficiency.

| Group | KEGG | | |
| --- | --- | --- | --- |
|  | Category | KEGG | Description |
| Bacteria  and  Archaea | Signaling and cellular processes | K01990 | Transport system ATP-binding protein |
|  |  | K06147 | ATP-binding cassette, subfamily B, bacterial |
|  |  | K01992 | Transport system permease protein |
|  |  | K02004 | Transport system permease protein |
|  |  | K02003 | Transport system ATP-binding protein |
|  | Genetic information processing | K03088 | RNA polymerase sigma-70 factor, ECF subfamily |
|  |  | K02529 | LacI family transcriptional regulator |
|  |  | K03657 | DNA helicase II / ATP-dependent DNA helicase PcrA [EC:3.6.4.12] |
|  | Lipid metabolism | K00059 | 3-oxoacyl-[acyl-carrier protein] reductase [EC:1.1.1.100] |
|  | Function unknown | K07133 | Uncharacterized protein |
|  | Signaling and cellular processes | K01990 | Transport system ATP-binding protein |
| Protozoa |  | K01992 | Transport system permease protein |
|  |  | K02004 | Transport system permease protein |
|  |  | K02003 | Transport system ATP-binding protein |
|  |  | K02015 | Iron complex transport system permease protein |
|  |  | K06147 | ATP-binding cassette, subfamily B, bacterial |
|  |  | K02016 | Iron complex transport system substrate-binding protein |
|  | Genetic information processing | K03088 | RNA polymerase sigma-70 factor, ECF subfamily |
|  |  | K02529 | LacI family transcriptional regulator |
|  | Signal transduction | K03406 | Methyl-accepting chemotaxis protein |

**Supplementary Figures**


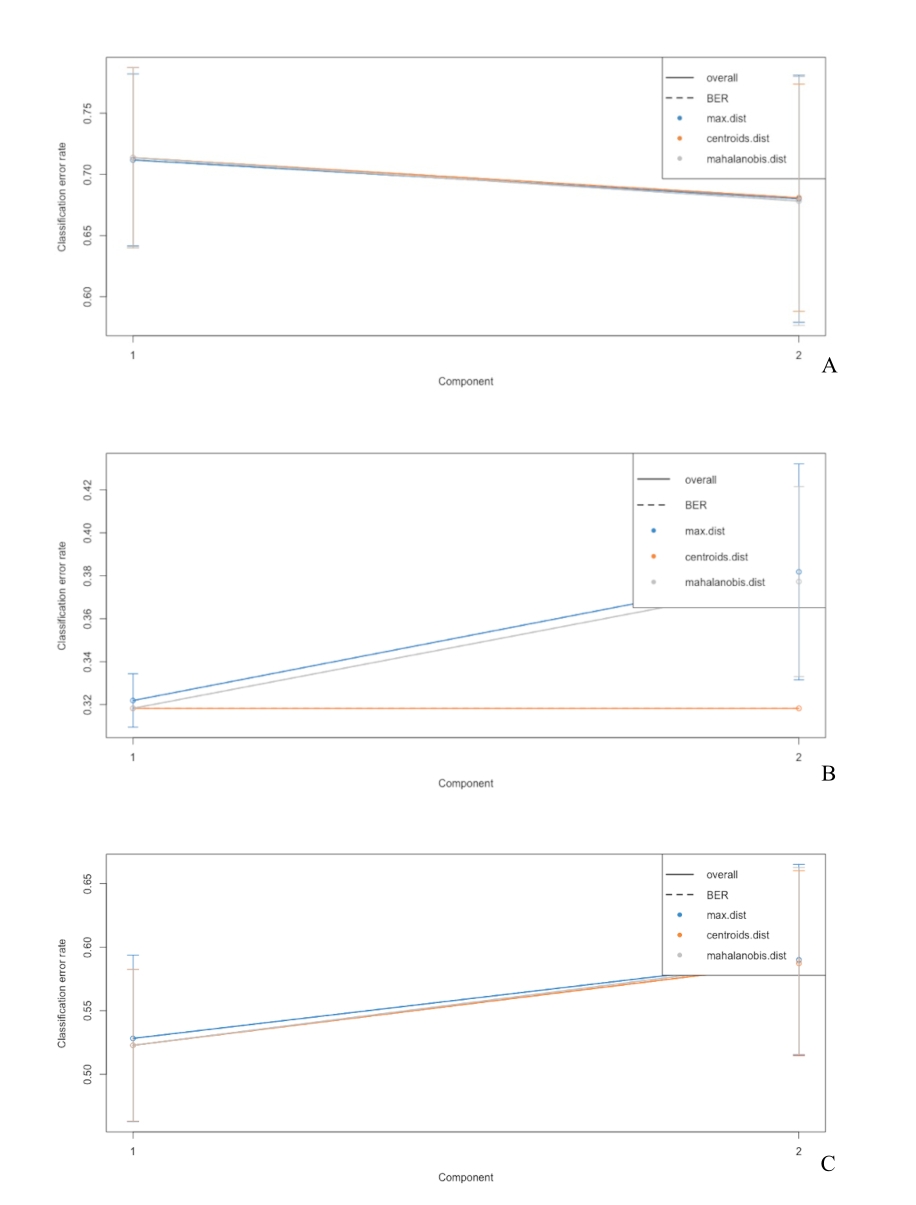


**Figure S1.** Classification performance per component for two predictions distances using repeated stratified cross-validation (10 × 5-fold Cross Validation). To estimate the classification error rate for the dataset, the distance metrics used for sPLS-DA (CLR transformed data) was the “centroids.dist”. **A.** Bacterial data; **B.** Archaeal data; **C.** Protozoal data.
